# Supplementary figures and images for: RNA sequencing (RNA-Seq) of lymph node, spleen, and thymus transcriptome from wild Peninsular Malaysian cynomolgus macaque (Macaca fascicularis)
Source: PeerJ. 2017 Aug 17;5:e3566. doi: 10.7717/peerj.3566 (PMC5563440; doi:10.7717/peerj.3566)

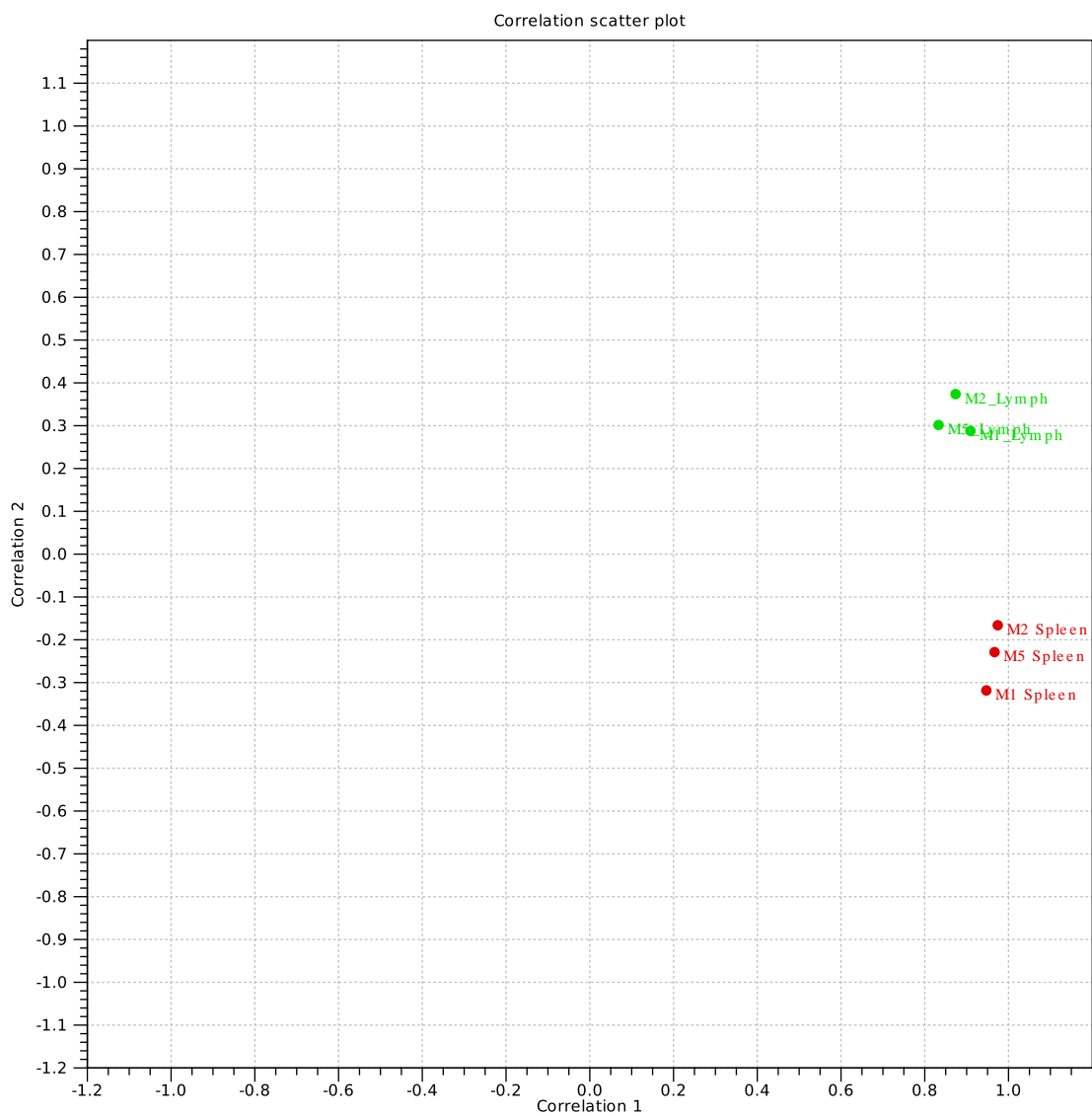

Supplement: Figure S1 [file peerj-05-3566-s001.pdf]

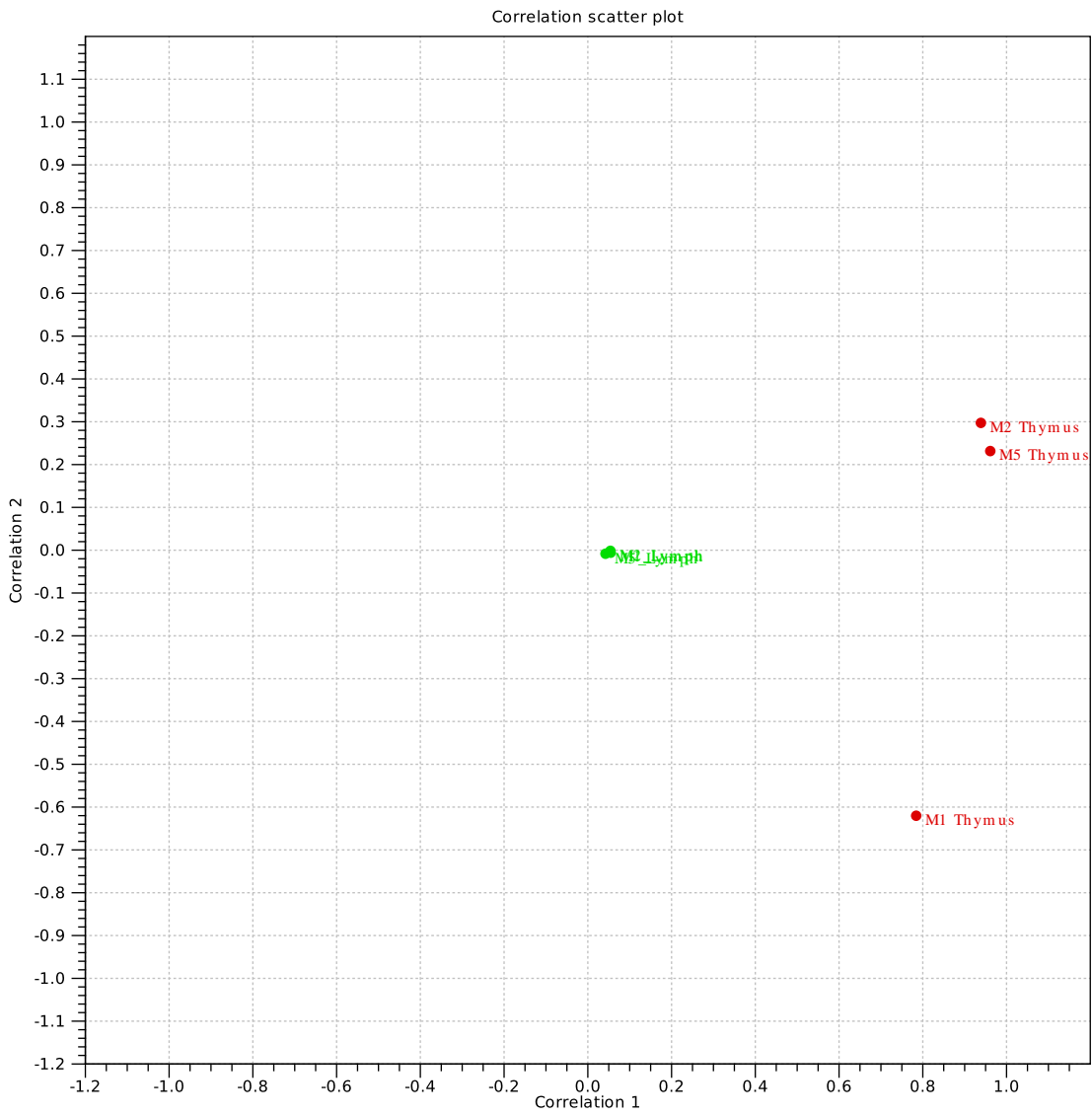

Supplement: Figure S2 [file peerj-05-3566-s002.pdf]

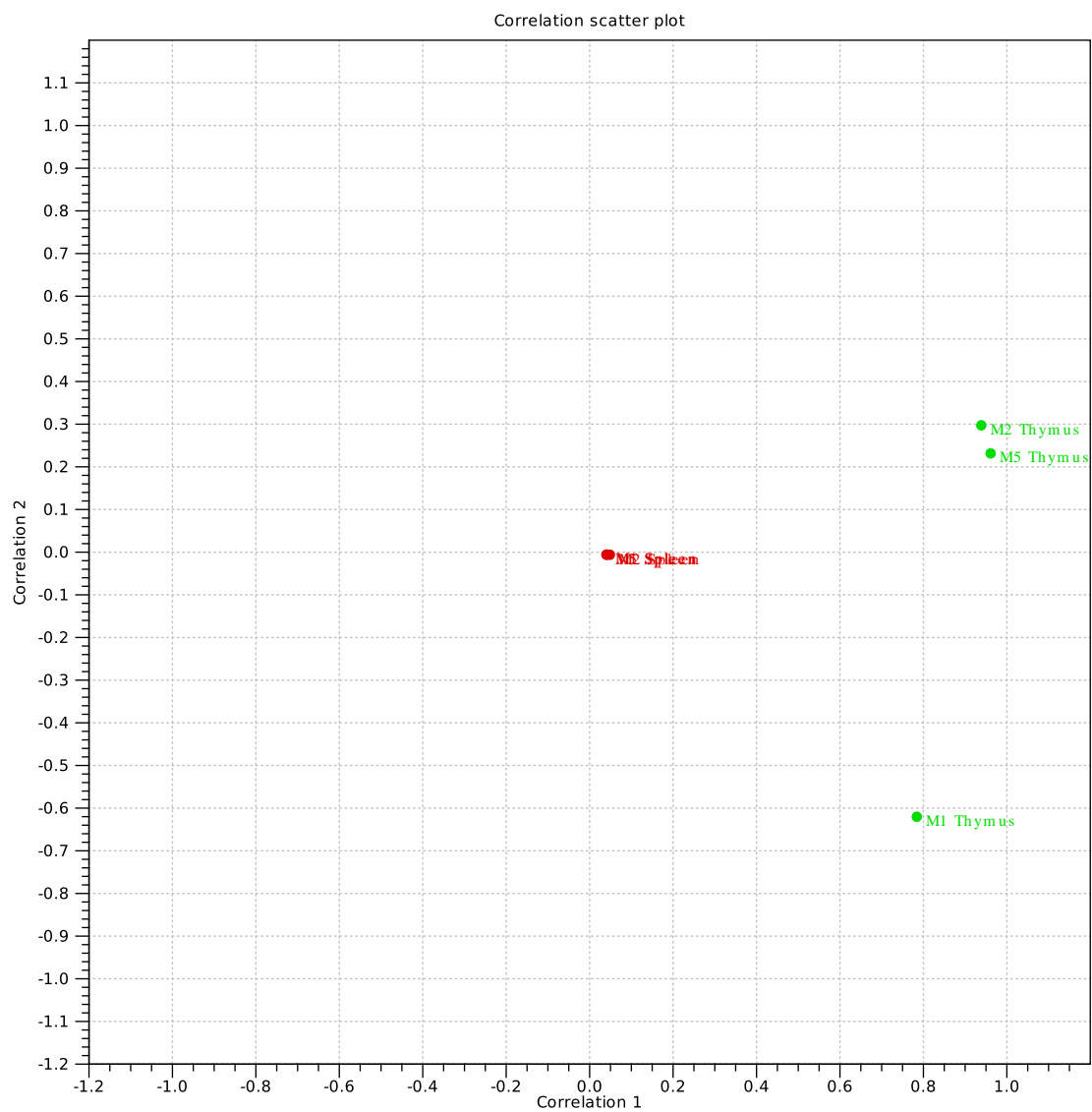

Supplement: Figure S3 [file peerj-05-3566-s003.pdf]

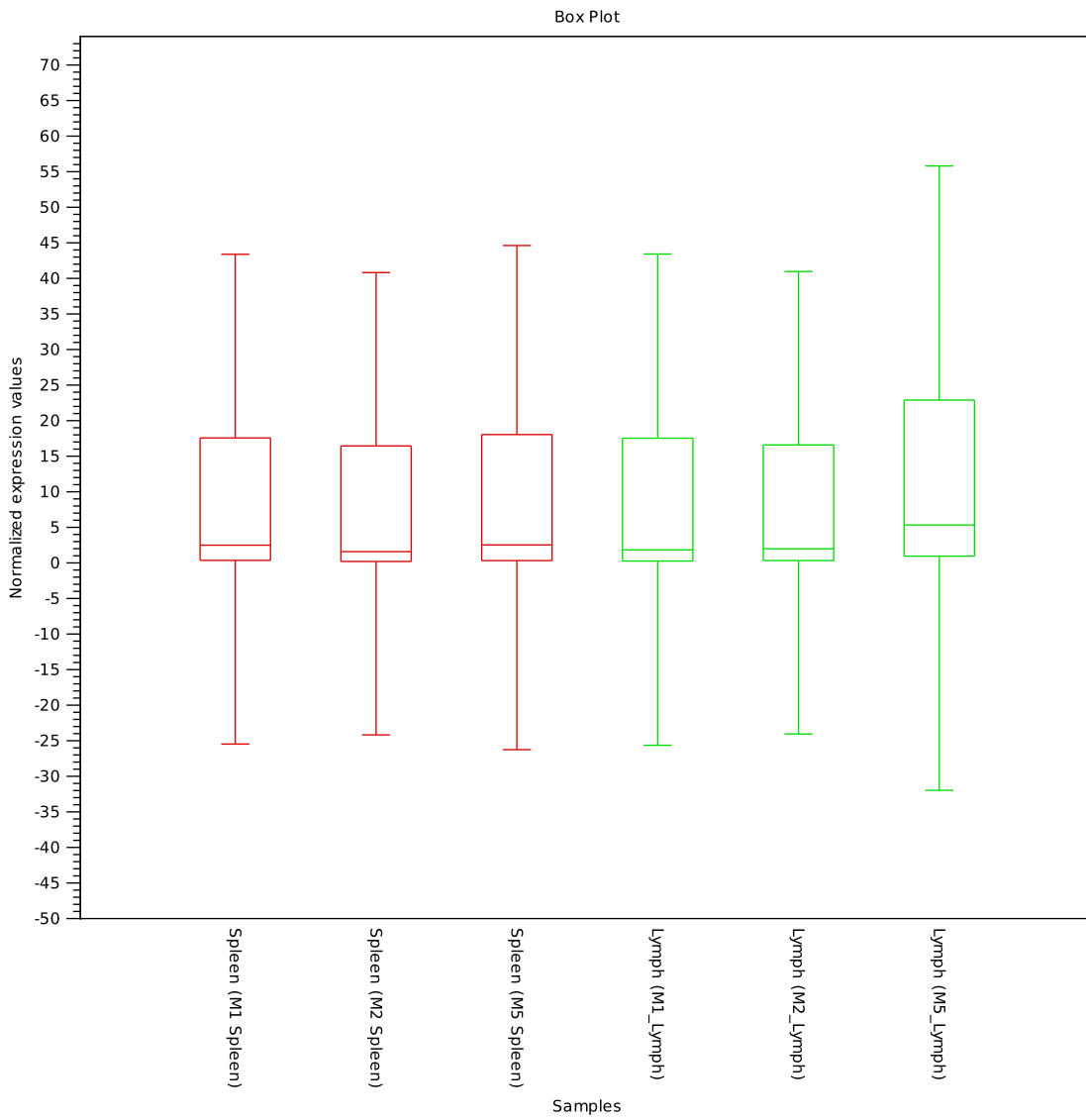

Y-axis: Normalised expression values in counts

Supplement: Figure S4 [file peerj-05-3566-s004.pdf]

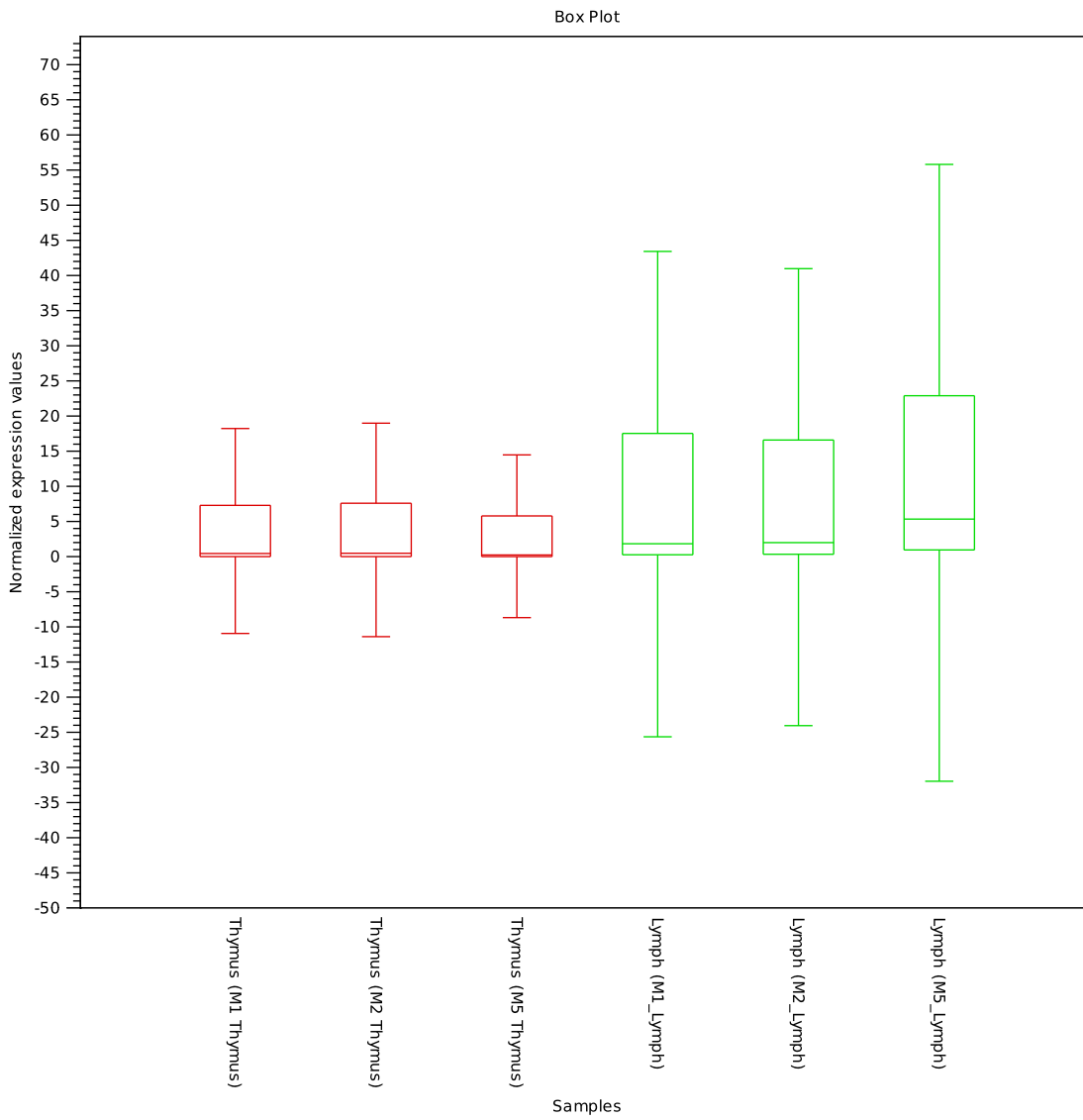

Y-axis: Normalised expression values in counts

Supplement: Figure S5 [file peerj-05-3566-s005.pdf]

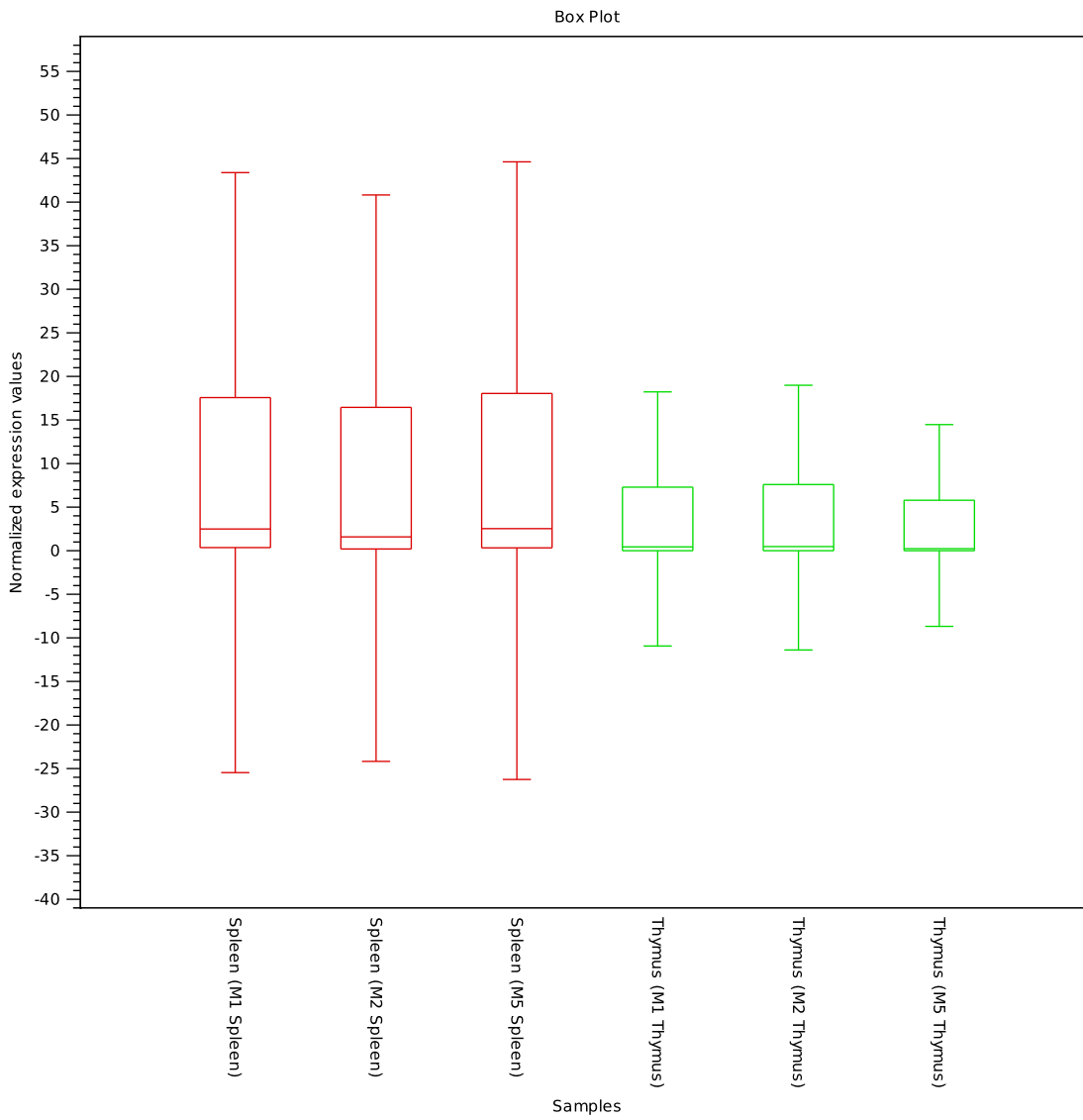

Y-axis: Normalised expression values in counts

Supplement: Figure S6 [file peerj-05-3566-s006.pdf]

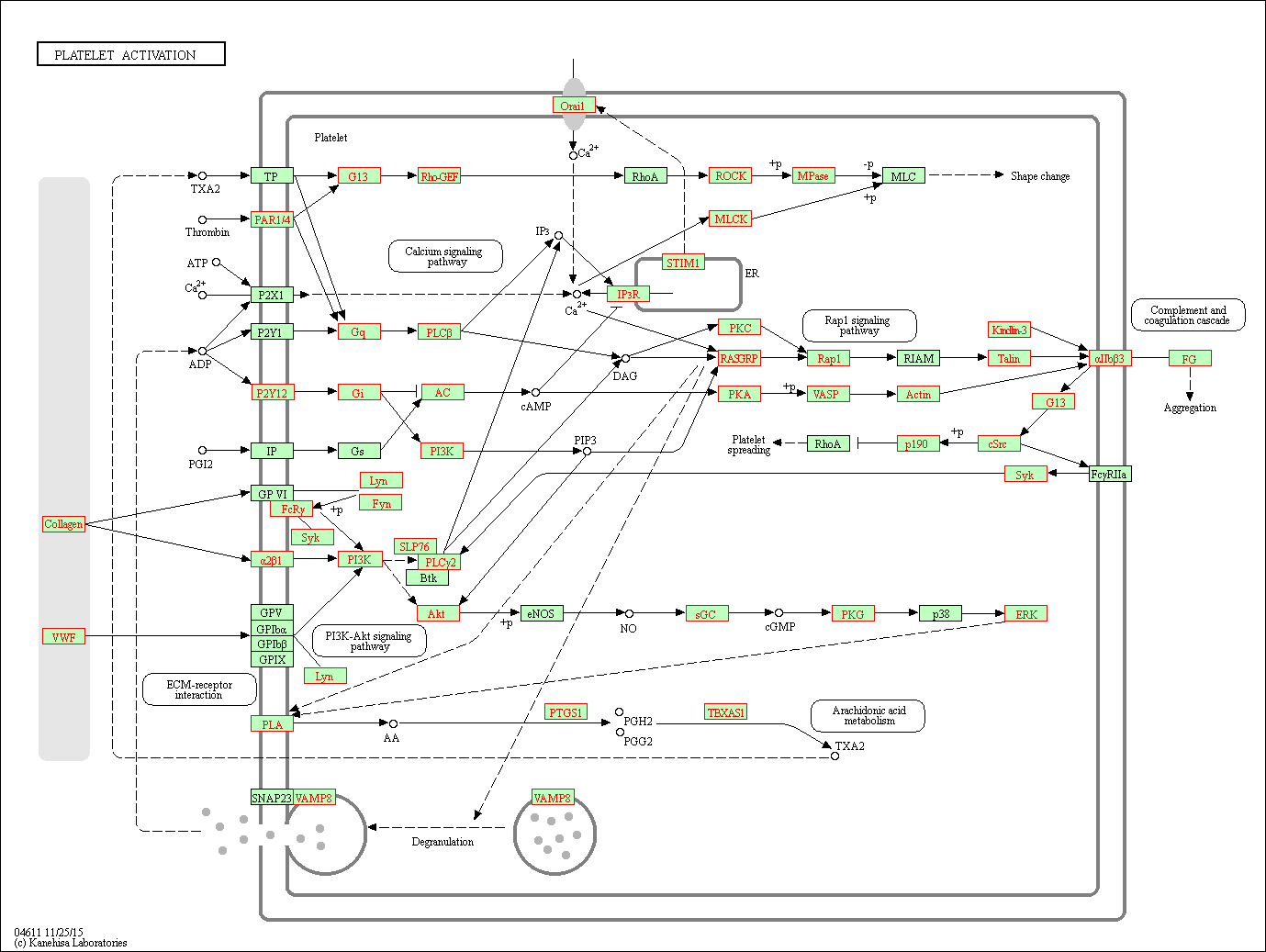

Supplement: Figure S7 [file peerj-05-3566-s007.png]

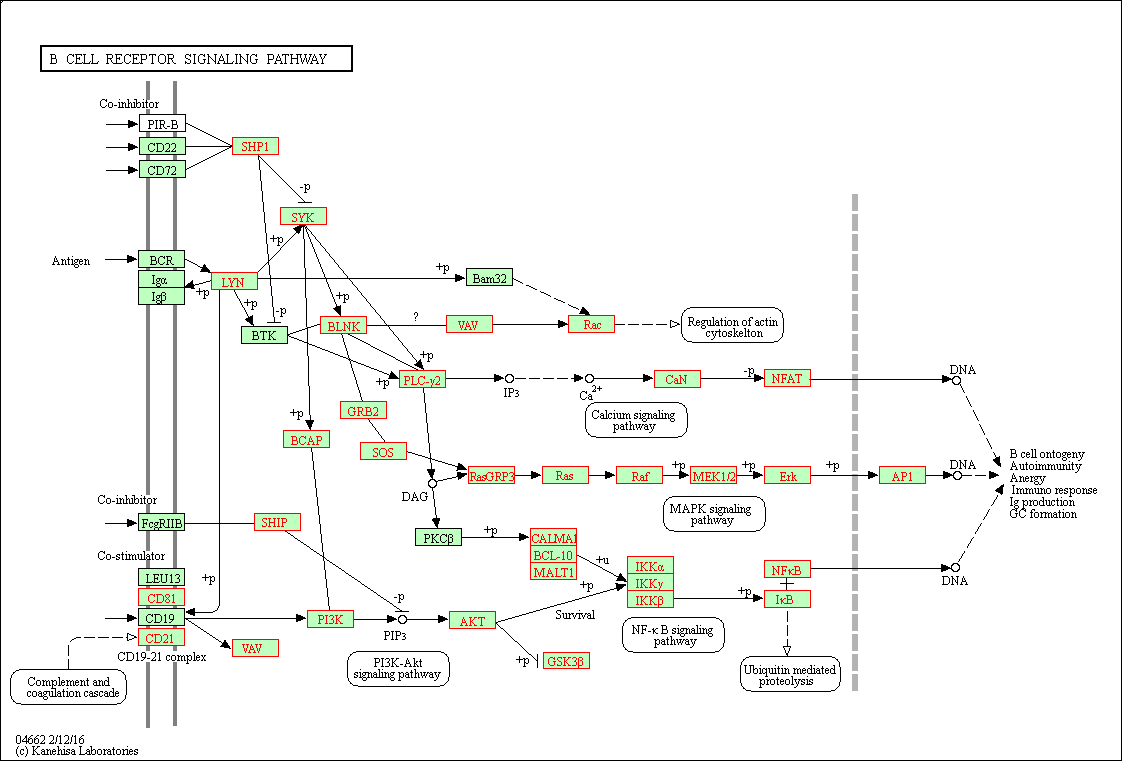

Supplement: Figure S8 [file peerj-05-3566-s008.png]

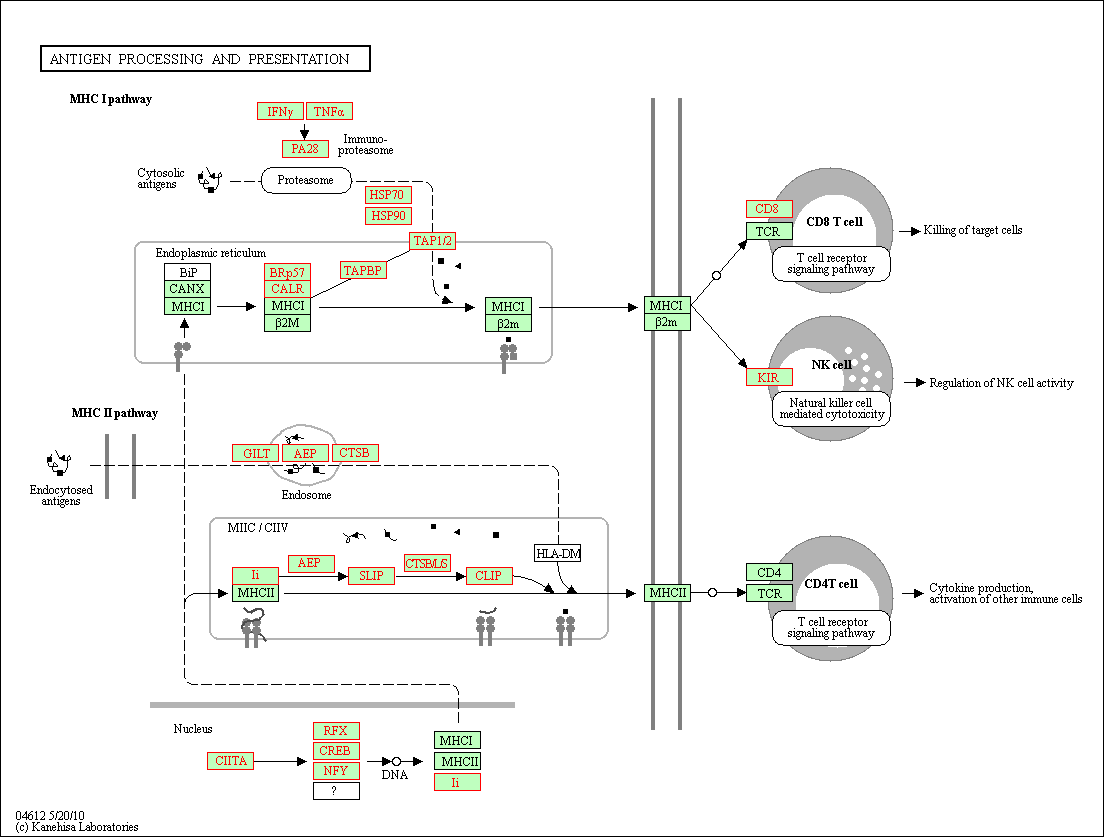

Supplement: Figure S9 [file peerj-05-3566-s009.png]

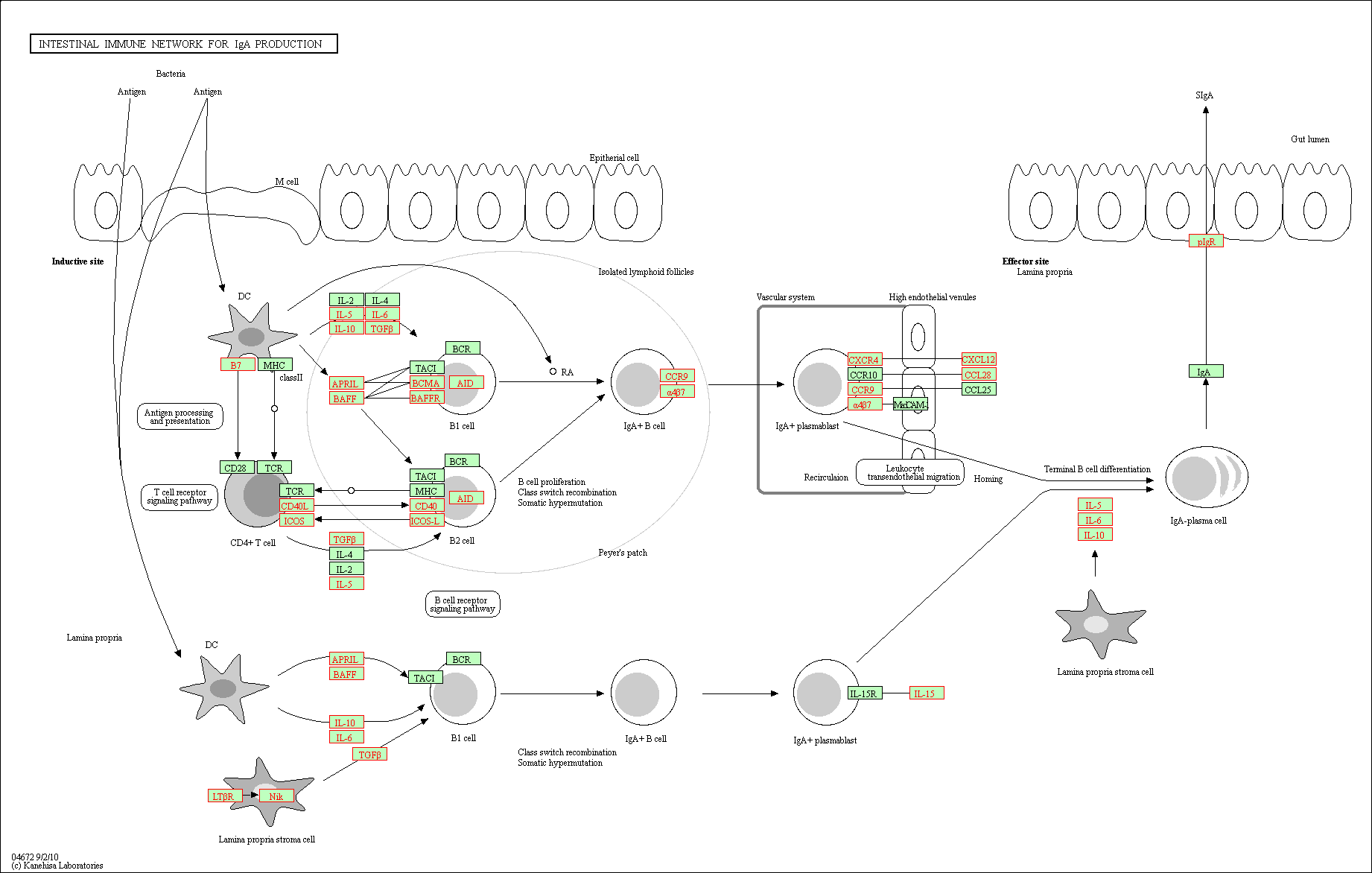

Supplement: Figure S10 [file peerj-05-3566-s010.png]

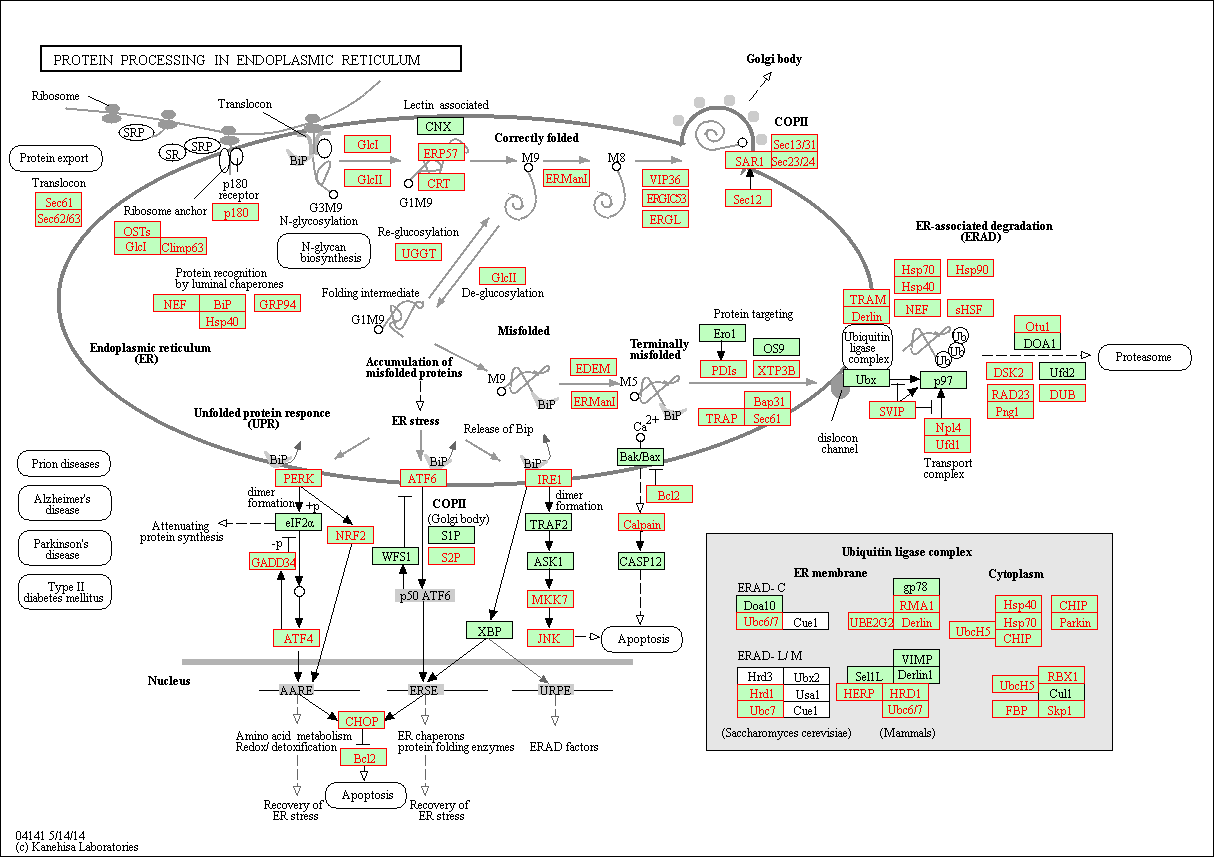

Supplement: Figure S11 [file peerj-05-3566-s011.png]

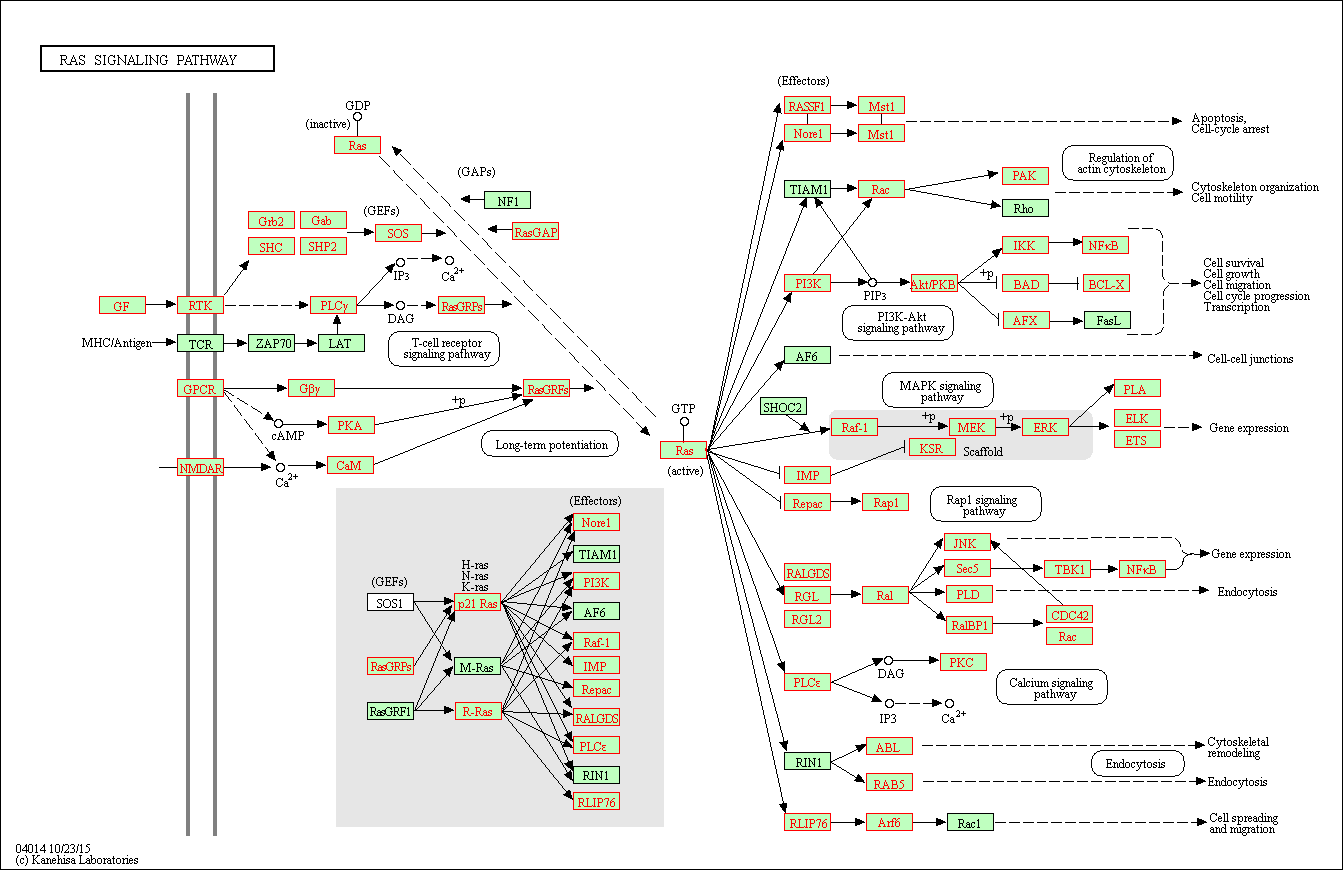

Supplement: Figure S12 [file peerj-05-3566-s012.png]

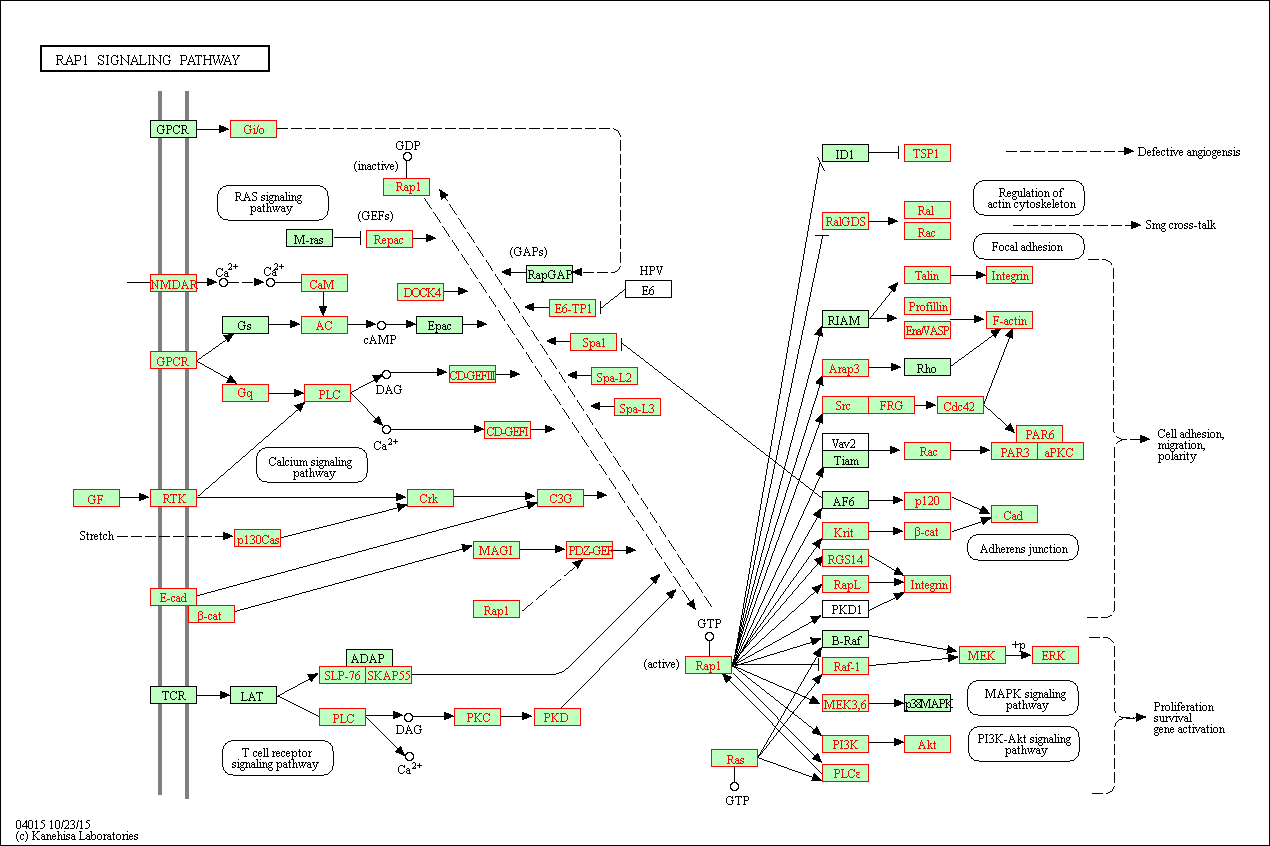

Supplement: Figure S13 [file peerj-05-3566-s013.png]

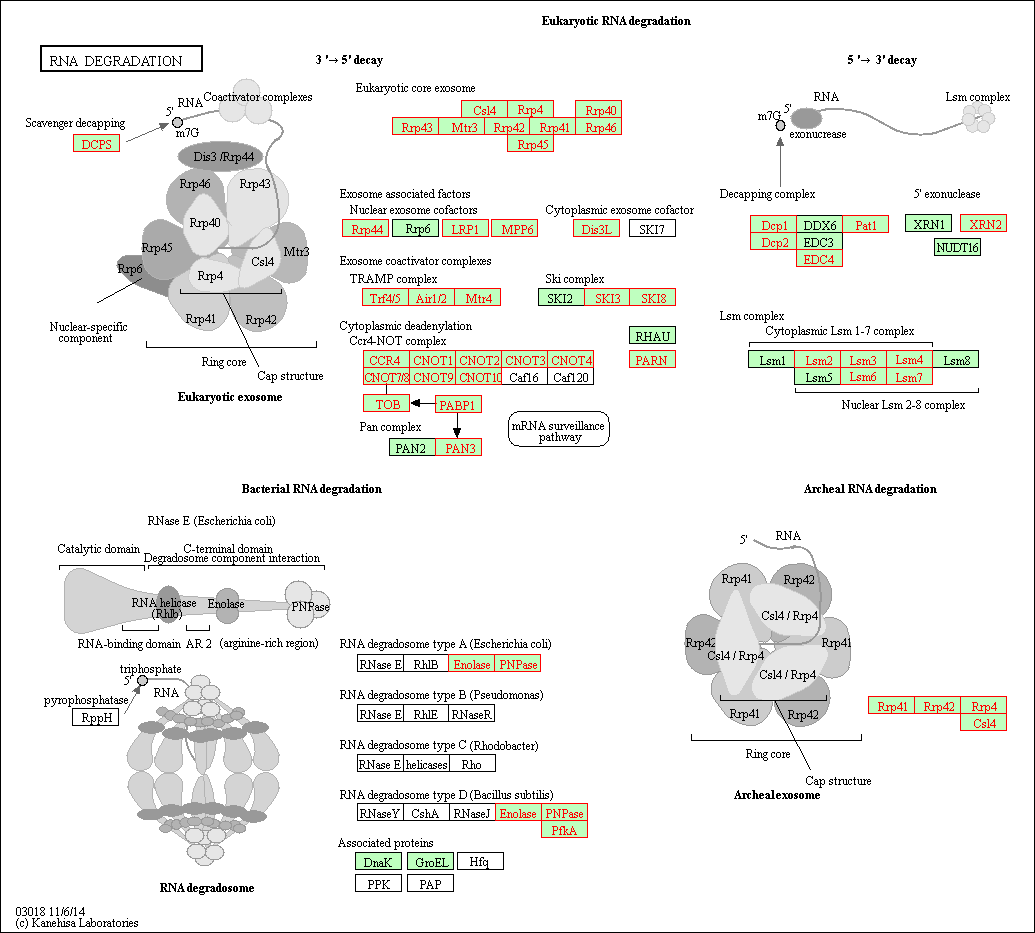

Supplement: Figure S14 [file peerj-05-3566-s014.png]

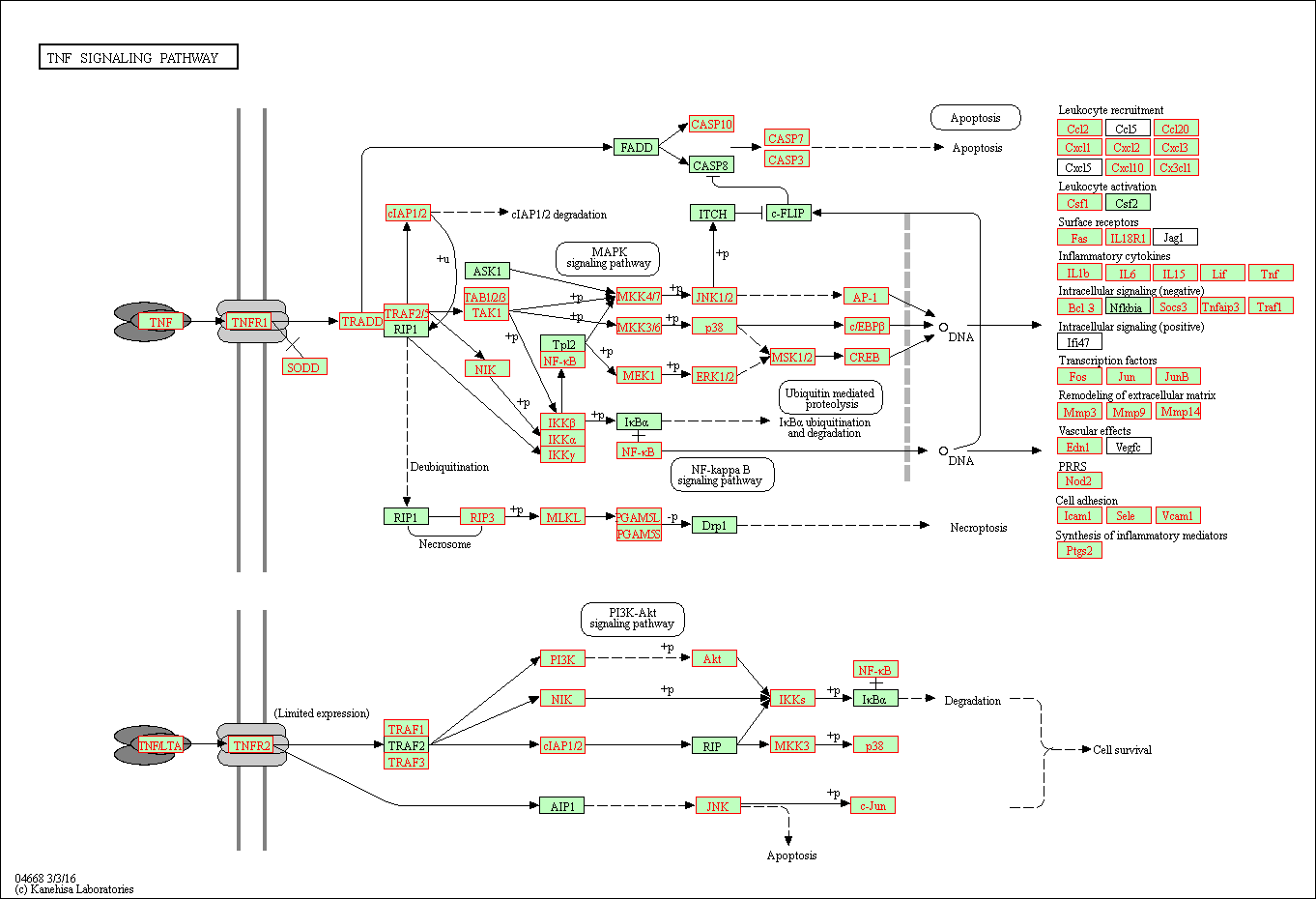

Supplement: Figure S15 [file peerj-05-3566-s015.png]

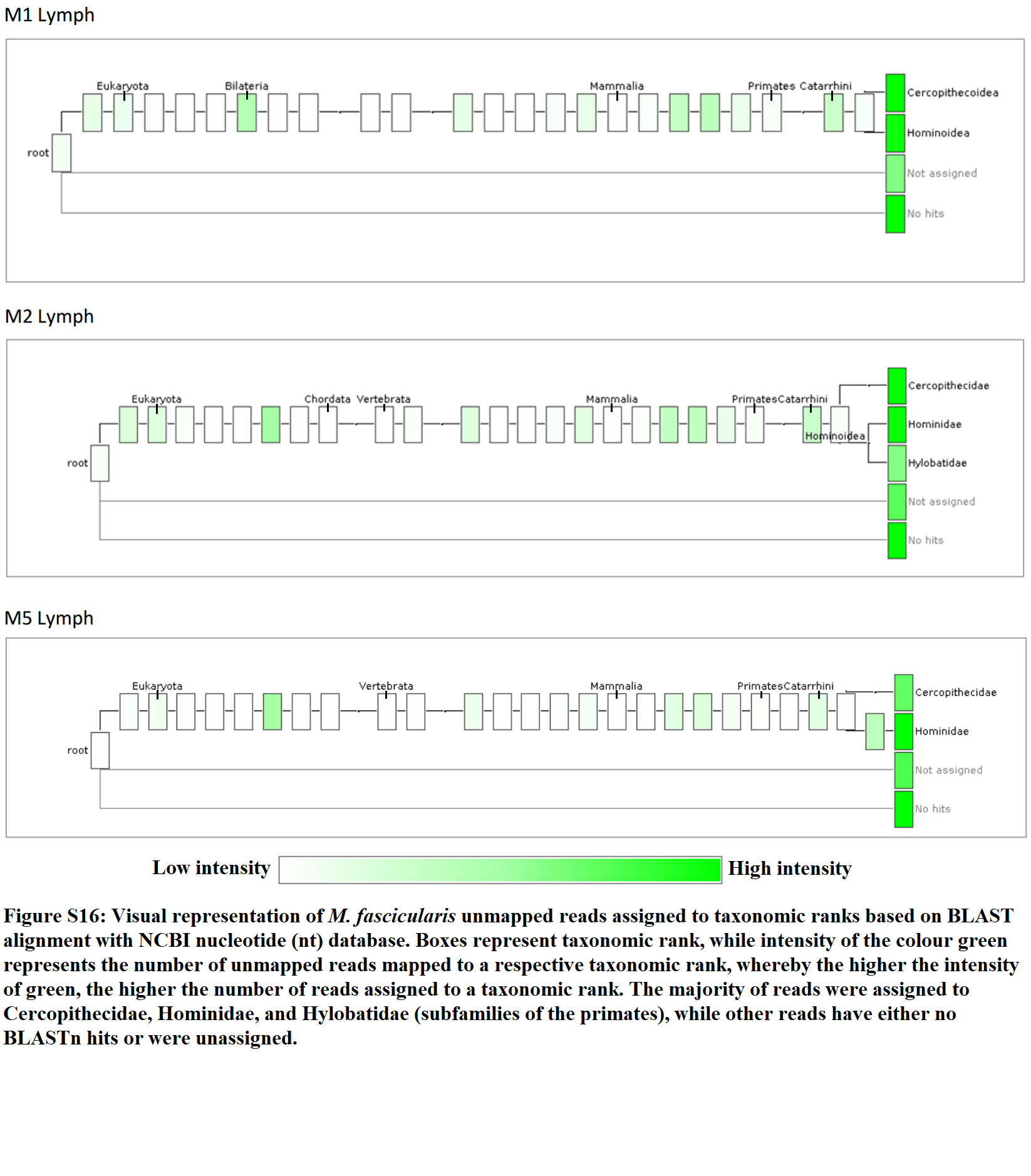

Supplement: Figure S16 [file peerj-05-3566-s016.png]
