# Supplementary material for: RNA sequencing (RNA-Seq) of lymph node, spleen, and thymus transcriptome from wild Peninsular Malaysian cynomolgus macaque (Macaca fascicularis)
Source: PeerJ. 2017 Aug 17;5:e3566. doi: 10.7717/peerj.3566 (PMC5563440; doi:10.7717/peerj.3566)
Supplement: Table S2 [file peerj-05-3566-s018.docx]

Table S2: Filtering and mapping statistics for each individual lymph node, spleen, and thymus libraries to *Macaca fascicularis* reference genome (GCF_000364345.1). Reads were mapped to gene regions only with a maximum number of hits for a read = 10. Only intact paired reads were taken into account when counting the fragments by type.

| **Sample** | **Sequencing Reads**  **(Before Trimming)** | **Sequencing Reads**  **(After Trimming)** | **Reads uniquely mapped to exon regions** | **Reads uniquely mapped to intron regions** | **Reads mapped to *M. fascicularis* reference in pairs (%)** | **Reads mapped to *M. fascicularis* reference in broken pairs (%)** | **Overall mapping percentage (%)** |
| --- | --- | --- | --- | --- | --- | --- | --- |
| M1 Lymph | 8,754,917 | 8,232,529 | 1,962,890 | 1,535,284 | 46.43 | 20.83 | 67.26 |
| M2 Lymph | 13,063,190 | 12,464,932 | 3,335,119 | 2,390,961 | 48.36 | 15.45 | 63.81 |
| M5 Lymph | 28,362,371 | 26,861,832 | 4,414,297 | 3,582,293 | 31.39 | 13.23 | 44.62 |
| M1 Spleen | 21,583,291 | 20,202,795 | 4,600,778 | 3,372,977 | 41.70 | 18.21 | 59.91 |
| M2 Spleen | 12,166,700 | 11,588,720 | 2,744,984 | 1,990,472 | 43.55 | 20.70 | 64.25 |
| M5 Spleen | 30,206,401 | 28,493,990 | 6,715,778 | 4,879,251 | 42.98 | 20.15 | 63.13 |
| M1 Thymus | 32,536,178 | 30,907,111 | 6,511,618 | 4,056,174 | 36.84 | 23.59 | 60.43 |
| M2 Thymus | 10,091,786 | 9,384,663 | 982,671 | 889,721 | 22.45 | 21.81 | 44.26 |
| M5 Thymus | 17,443,244 | 16,291,578 | 2,025,947 | 1,728,337 | 26.51 | 22.31 | 48.82 |
